# Classifying musical reading expertise by eye-movement analysis using machine learning

**DOI:** 10.3389/fcogn.2024.1417011

**Published:** 2024-08-30

**Authors:** Véronique Drai-Zerbib, Manon Ansart, Clément Grenot, Bénédicte Poulin-Charronnat, Joris Perra, Thierry Baccino

**Affiliations:** Laboratoire d'Étude de l'Apprentissage et du Développement, LEAD – CNRS UMR5022, Université de Bourgogne, Dijon, France

**Keywords:** sight reading, expertise, musicians, classification, machine learning, SVM, eye movements

## Abstract

Music reading is the key to literacy for musicians in the Western music tradition. This high-level activity requires an efficient extraction of the visual information from the score to the current needs of the execution. Differences in eye movements between expert and non-expert musicians during music reading have been shown. The present study goes further, using a machine learning approach to classify musicians according to their level of expertise in analyzing their eye movements and performance during sight-reading. We used a support vector machine (SVM) technique to (a) investigate whether the underlying expertise in musical reading could be reliably inferred from eye movements, performance, and subjective measures collected across five levels of expertise and (b) determine the best predictors for classifying expertise from 24 visual measures (e.g., the number of progressive fixations, the number of regressive fixations, pupil size, first-pass fixations, and second-pass fixations), 10 performance measures (e.g., eye–hand span, velocity, latency, play duration, tempo, and false notes), and 4 subjective measures (perceived complexity and cognitive skills). Eye movements from 68 pianists at five different levels of music expertise (according to their level in the conservatory of music—from first cycle to professional) were co-registered with their piano performance via a Musical Instrument Digital Interface, while they sight-read classical and contemporary music scores. Results revealed relevant classifications based on the SVM analysis. The model optimally classified the lower levels of expertise (1 and 2) compared to the higher levels (3, 4, and 5) and the medium level (3) compared to higher levels (4 and 5). Furthermore, across a total of 38 measures, the model identified the four best predictors of the level of expertise: the sum of fixations by note, the number of blinks, the number of fixations, and the average fixation duration. Thus, efficiently classifying musical reading expertise from musicians' eye movements and performance using SVM is possible. The results have important theoretical and practical implications for music cognition and pedagogy, enhancing the specialized eye and performance behaviors required for an expert music reading.

## Introduction

Music reading requires extracting visual information from the musical score, tailored to the immediate demands of performance, and is the hallmark of expert musicians. In particular, music sight-reading is a highly demanding cognitive task, as it consists of reading and performing a piece of music at first sight or after very little preparation, coordinating visual, auditory, and motor processing while respecting the temporal constraints inherent to the composition. During sight-reading, visuospatial symbols (notes and codification placed on the staff) are translated into sounds, attention is continuously directed toward the forthcoming notes (Rayner, 1998), and an integrative multimodal information processing is required to efficiently process information from auditory, visual, and motor modalities (Drai-Zerbib and Baccino, 2005, 2014, 2018; Stewart et al., 2003). Moreover, the effective reading of various musical scores in the tonal music tradition, commonly associated with classical music, is a skill mastered by expert musicians. This process involves prioritizing several musical elements from the score, such as notes, rhythm, harmony, tonal rules, dynamics, and musical form, within the context of the musical style. Acquiring expertise in reading and interpreting musical notation is foundational to attaining expertise in music. Understanding the development of a young musician's cognitive system over the years of learning to achieve expertise in their discipline is a scientific and pedagogical challenge. Whether musicians are composers, singers, instrumentalists, or conductors, their activity involves deciphering, reading, writing, composing, transcribing musical phrases, interpreting musical language, and musical reading. These activities engage knowledge related to the written code and its reference framework (such as period, style, musical form, composer, etc.). Generally, reading, an activity not genetically programmed, must find its place in the reader's brain. While language naturally establishes itself during the early years of a neurotypical child, reading requires intensive learning and practice. Once established, reading leaves an anatomical signature in the brain of the expert reader, inducing cerebral structural modifications (Carreiras et al., 2009). Musical reading, as text reading, necessitates learning and practice to become an expert skill. Therefore, elucidating how the cultural object of musical reading is integrated into the cognitive system is crucial in facilitating the efficient real-time processing of multimodal information to achieve a high level of sight-reading performance. Comparing different levels of musicians and highlighting the key behaviors that reflect the development of musical expertise over the years of learning is a perfect way to understand the construction of this expertise and its fundamental cognitive markers. Identifying the most relevant ocular and behavioral indicators of expertise levels is essential in this endeavor.

Using an eye-tracking method to investigate the cognitive processes underlying music reading has revealed significant inter-individual differences associated with musical expertise (Drai-Zerbib and Baccino, 2014; Drai-Zerbib et al., 2012; Perra et al., 2022). Many studies in this field have examined such differences through the lens of distinct memory encoding and retrieval strategies (Drai-Zerbib, 2016; Drai-Zerbib and Baccino, 2018) related to developing an expert memory, such as the chunking theory (Chase and Simon, 1973; Ericsson and Chase, 1982; Maturi and Sheridan, 2020; Waters et al., 1998) and the long-term working memory (Ericsson and Kintsch, 1995) applied in the domain of music reading (Drai-Zerbib and Baccino, 2005, 2014, 2018; Williamon and Valentine, 2002). In line with the principles characteristic of expert memory, expertise in music reading results in structural information processing involving meaningful encoding (organizing information), retrieval structures previously built in long-term memory (LTM), and an acceleration of information encoding and retrieval with practice. Therefore, expert memory empowers musicians to leverage an expanded working memory within LTM via retrieval cues. Their network of knowledge in LTM enables them to efficiently recognize frequent patterns or a *chunk* of notes, chords, arpeggios, or rhythms (Sheridan et al., 2020; Waters et al., 1997) and benefit from higher level processing, hierarchically linking elements to the musical structure of a score (Aiello, 2001; Drai-Zerbib, 2016; Drai-Zerbib and Baccino, 2005; Perra et al., 2024; Williamon and Valentine, 2002). As a result, the expert draws on prior knowledge to encode the presented elements in a meaningful way and store them by grouping them into LTM. This is what enables expert musicians to exhibit shorter fixation durations (Drai-Zerbib and Baccino, 2005, 2014, 2018; Drai-Zerbib et al., 2012; Goolsby, 1994; Penttinen et al., 2013; Perra et al., 2022; Waters and Underwood, 1998; Waters et al., 1997), fewer number of fixations (Drai-Zerbib and Baccino, 2014; Waters et al., 1997), and an increase in eye–hand span (EHS; Furneaux and Land, 1999; Penttinen et al., 2015; Perra et al., 2021; Sloboda, 1974; Truitt et al., 1997) compared to non-experts. Thus, sight-reading expertise involves a higher processing speed and a more effective extraction of information from the score. Eye movements can therefore indicate differences in expertise. In addition, eye movements reveal the progression of music reading skills in novice musicians, who gradually reduce the duration of their fixations on a score with training (Penttinen and Huovinen, 2011). The musical performance itself is evolving with expertise, with an increase in accuracy and chosen tempo when sight-reading (Drake and Palmer, 2000; Truitt et al., 1997; Zhukov et al., 2019).

Comparing expert and non-expert musicians, previous studies have shown that eye movements in sight-reading evolve with the development of musical expertise and may reflect the degree of elaboration of expert memory structures developed over years of learning and practice (Drai-Zerbib and Baccino, 2018; Penttinen and Huovinen, 2011; Perra et al., 2024). Our present study aims to go further by using an advanced machine learning technique to classify musicians according to their level of expertise by analyzing their eye movements synchronized with their playing behavior during a score sight-reading. This classification is a supervised learning process, as the machine learning process is based on a set of observations that have previously been correctly identified. The principle is to train the algorithm (the machine) to perform a specific task using a substantial amount of provided examples (previously collected data) belonging to one or more categories to subsequently categorize and separate the data into multiple classes. A variety of multivariate pattern analysis (MVPA) techniques, such as support vector machines (SVMs), naïve Bayes, or *k*-nearest neighbors, are capable of classifying different profiles. These techniques establish classification procedures and are part of machine learning methods used to identify to which category (subpopulation) a new observation belongs, based on a data set containing observations whose category membership is known. The SVM (Cortes and Vapnik, 1995) has been extensively studied, is one of the most reliable classification techniques (Guyon et al., 2002) and is being used more and more in cognition. The SVM is a supervised linear classification algorithm. Its primary function is to separate data using a hyperplane to maximize the distance between points belonging to different classes. Consequently, SVM divides a data set into several classes or groups based on their defining values, ensuring that the distance between distinct groups of data and that the margins between them are maximized. SVMs have been shown to be one of the best supervised learning methods in various applications (Cervantes et al., 2020). SVM has been successfully applied to eye movements, for instance, to predict a reader's literacy level by analyzing their eye movements during text reading (Lou et al., 2017), classify scan paths in reading, predict a reading and text comprehension profile (Makowski et al., 2019), and infer the tasks (pseudo-reading, scene search, and scene memorization) that viewers were engaged in Henderson et al. (2013). In an exploratory study, MVPAs were successfully used to classify expert and non-expert musicians based on their visual performance (e.g., fixation duration, saccade amplitude, and pupil dilation) while reading a musical score (Baccino and Drai-Zerbib, 2015). However, to the best of our knowledge, so far, no study has used SVM techniques to identify musicians' reading levels across five levels of expertise based on their eye movements and performance during sight-reading of music.

Using SVM analysis, the current study aims to (a) investigate the extent to which the level of expertise in reading music notation can be reliably inferred from eye movements, performance, and subjective measures across five levels of expertise and (b) identify the most relevant predictors for classifying levels/groups of expertise from visual measures (e.g., number of progressive fixations, number of regressive fixations, pupil size, first-pass fixations, and second-pass fixations), performance measures [e.g., EHS, percentage of incorrect notes per area of interest (AOI), tempo, play duration, and velocity], and subjective measures (perceived complexity and cognitive skills).

## Method

### Participants

In total, 68 participants, including students, teachers, and professional musicians from French music conservatories, were recruited for the study. They were categorized into five groups based on their musical training at the music conservatory: 15 participants were students in the first cycle (*M*_age_ = 11.47 years, *SD* = 1.69), 15 were in the second cycle (*M*_age_ = 14.00 years, *SD* = 3.14), 14 were in the third cycle (*M*_age_ = 20.14 years, *SD* = 3.63), eight were from the Classe Préparatoire à l'Enseignement Supérieur (CPES) equivalent to a college level in the international system (*M*_age_ = 21.63 years, *SD* = 6.80), and 16 were from the Conservatoire National Supérieur de Musique (CNSM) or professional musicians (*M*_age_ = 38.44 years, *SD* = 12.74). Those different levels of expertise will be thereafter, respectively, named levels 1, 2, 3, 4, and 5. Noteworthy is that the CPES group (level 4 of expertise) had only eight participants, in contrast to the other levels, which had at least 14 participants, as the CPES pianist population was rather difficult to recruit. However, we previously conducted statistical analyses to investigate the relevance of the five experimental groups. *k*-means analyses based on the number of fixations and the chosen tempo revealed that the 68 musicians could be classified into five distinct expertise groups, *F*_(4, 63)_ = 41.701, *p* < 0.001. The results indicated also that the CNSM students, who were at the end of their study in this higher education institution for musicians (already semi-professionals) and professional musicians could be considered to belong to the same group (level 5). Participants had to be pianists completing a music conservatory cycle or professional to be included in the experiment. All participants had normal or corrected-to-normal vision. Incentives for participation included a gift card worth €15.

### Material

**Figure 1:**
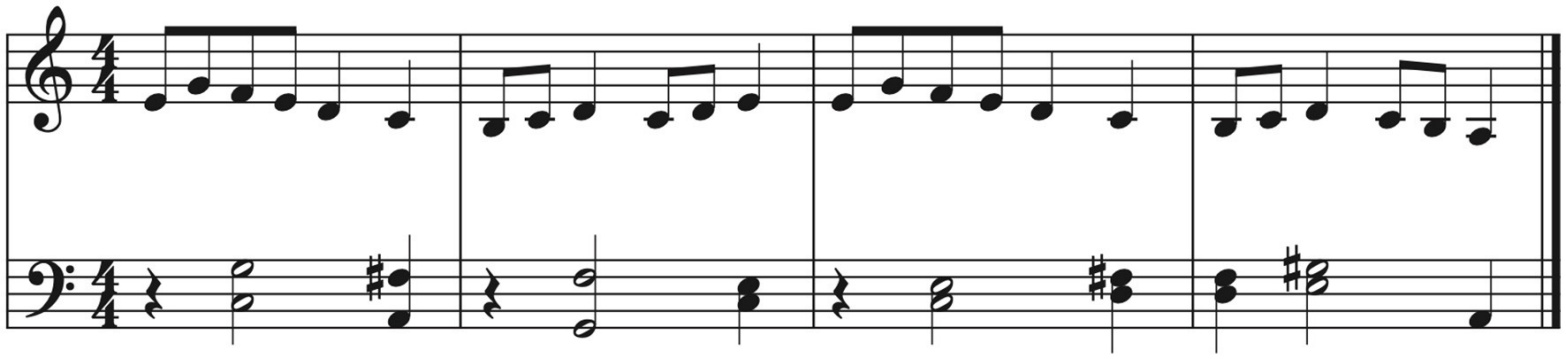
Example of a classical score.

**Figure 2:**
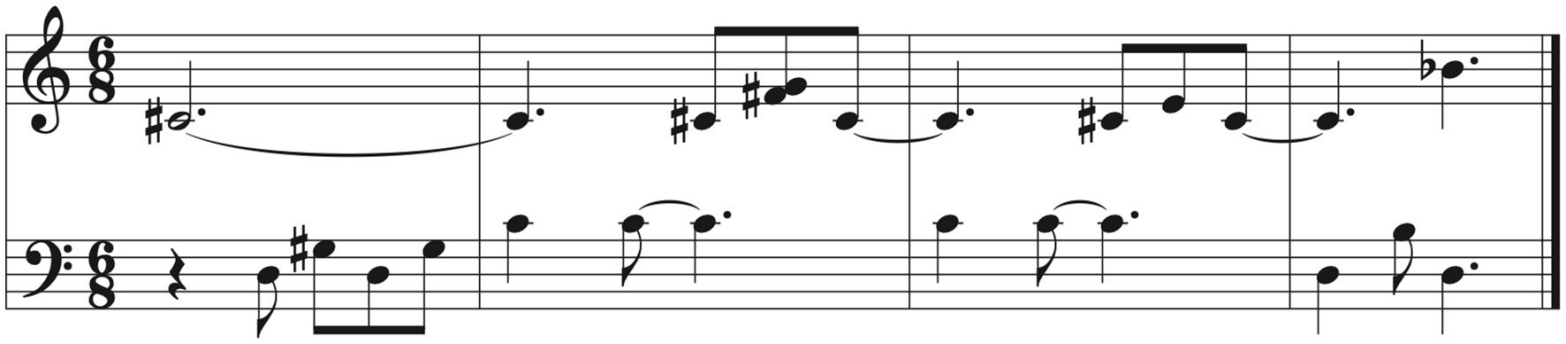
Example of a contemporary score.

The material comprised 68 dual-staff excerpts, each consisting of four bars, extracted from piano compositions. The excerpts were carefully selected at different difficulty levels and types of musical texture to represent the ecological scores encountered in the participants' regular practice routines (see Appendix 1). This selection ensured that the scores were adapted to the students' levels and were sufficiently challenging for more advanced musicians. We sought to train our SVM on classical and contemporary scores. As contemporary scores are less practiced during music education and more demanding (in terms of mental workload, in particular for lower levels), we decided to include a reduced number of contemporary scores compared to classical scores. Thus, 43 of the selected excerpts were in the classical music style, respecting the rules of the Western tonal system, and 25 were in the contemporary music style, from the atonal music repertoire (Figures 1, 2). The full material (68 excerpts) was presented to the higher levels (levels 3–5), whereas 34 excerpts (23 composed in the classical music style and 11 composed in the contemporary music style) were presented to lower levels (1–2). All excerpts were generated using the Final^TM^ music software. They were displayed on a 17″ screen with a resolution of 1920 × 1080 pixels. The presentation order of the excerpts was randomized across the participants.

### Apparatus

**Figure 3:**
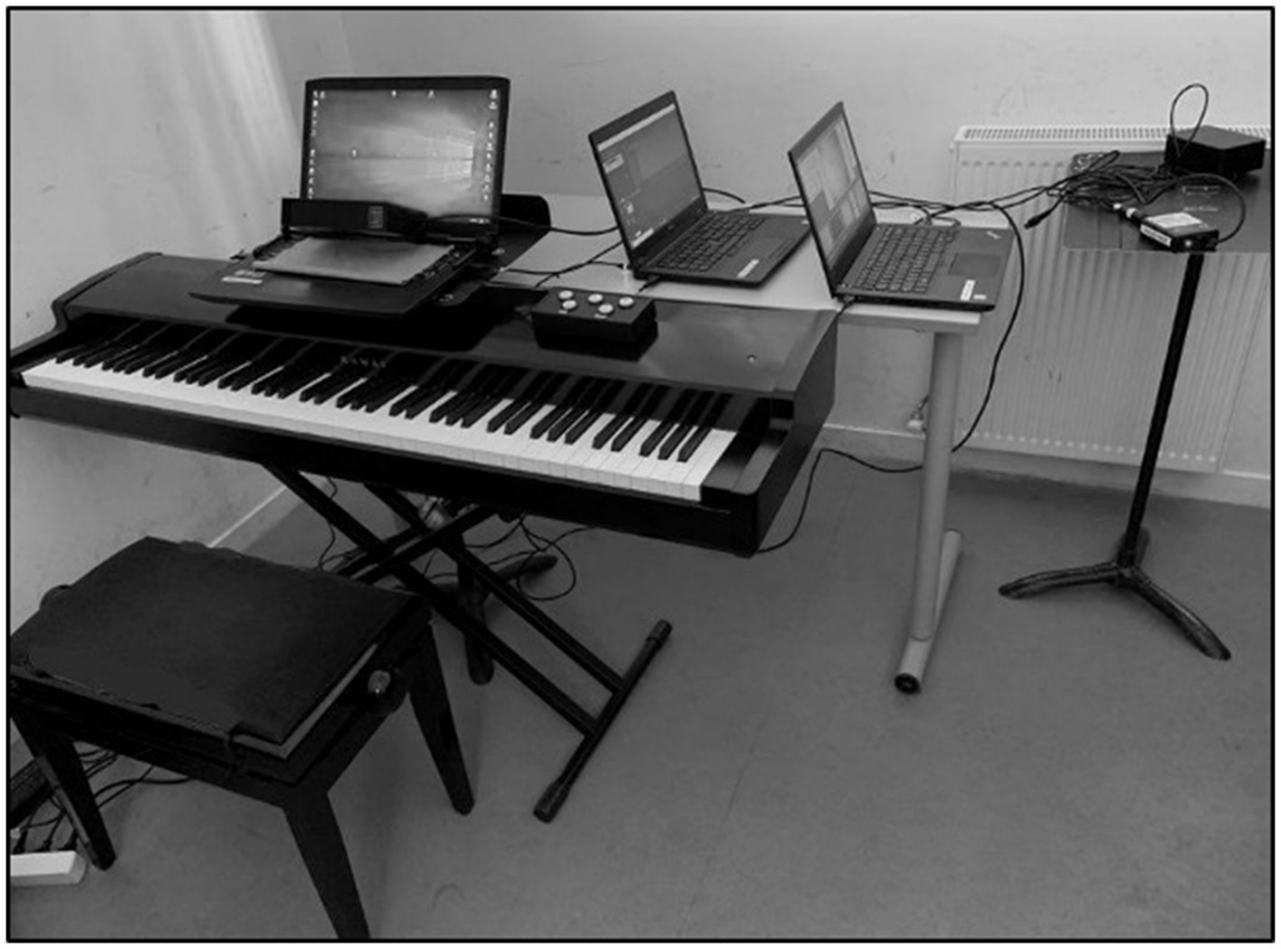
Experimental setup.

Eye movements were recorded during sight-reading with an EyeLink Portable Duo 1000 eye tracker (SR Research™). Participants sat 60 cm away from the monitor. The experiment was controlled with Experiment Builder software (SR Research). Both eyes were tracked with a sampling rate of 1,000 Hz. To make the experimental conditions as ecological as possible, the musicians were not constrained by a chin strap, as this type of eye tracker allowed for free head movement. To ensure the best tracking quality of the pupil diameter, measurements were taken under constant luminance (light coming from the monitor) and illuminance (artificial ambient illumination; Benedetto et al., 2014). Moreover, all the stimuli (four measures) were constantly presented at the center of the screen; after gazing, a cross of fixation was presented at the location of the stimulus), avoiding any important rotation of the musicians' eyes. Thus, any potential concern of the pupil size measures was minimal. The recording of eye movements was synchronized with the recorded piano performance using a Musical Instrument Digital Interface (MIDI), whereby the input from the piano (KAWAI VPC1 with an RM3 Grand II with wooden key action) was transmitted to Reaper^TM^ software on a separate computer. The transmission of the trigger from the experiment builder software to the reaper software (MIDI file) and the eye recording (.evs file) occurred without any potential delay between the signals. On this basis, analyzing the EHS is possible by comparing the position of the ocular fixation and the note played at a given time. The experimental setup is presented in Figure 3.

### Pretests

To evaluate the different psychological abilities of the musicians and include those subjective measures into the SVM, three cognitive pretests were individually administered to the participants to assess their working memory and processing speed capacities. The Wechsler Intelligence Scale for Children (WISC-V) or Weschler Adult Intelligence Scale (WAIS-IV) Digit Span subtest, with digit span forward, digit span backward, and sequencing (ascending order) tests, assessed auditory recall, short-term memory, and working memory (auditory–verbal working memory); the Coding subtest assessed psychomotor speed, visuomotor speed, capacity to process a new code (processing speed); and the Corsi Block tapping test with right-side-down tests assessed visuospatial working memory.

### Posttests

As expertise in music reading is associated with an improved ability to handle complexity (Perra et al., 2024), the perceived complexity assessment was collected to be included in the SVM. After sight-reading each score, the musicians were required to assess the perceived complexity of the musical score on a Likert scale ranging from 1 (*very easy*) to 5 (*very difficult*). The musicians were also instructed to assess whether they already knew the score to ensure that they were not familiar with the proposed material. In addition to providing crucial subjective data, these two questions enabled the participant to reflect on their previous sight-reading score.

### Procedure

The data acquisition was conducted in three music conservatories, each time using the same experimental setup in a quiet room of the music conservatory. After the written instructions were presented, the participants completed a questionnaire regarding their musical background and underwent cognitive pretests. Then, the participants settled comfortably to the piano to be ready to play in front of the eye tracker. They were instructed to engage in self-paced sight-reading at their chosen tempo. Participants at higher levels (levels 3–5) were presented with the full material (68 excerpts), while participants at lower levels (levels 1–2) were presented with their level-related 34 excerpts (23 in the classical music style and 11 in the contemporary music style) related to their level of expertise in reading. A 9-point calibration procedure was conducted at the outset of the experiment. An average spatial error of up to 0.5° was deemed acceptable with a maximum allowable spatial error set to 1° of visual angle. After a training trial, eye movements were recorded. Before each excerpt, the participant had to fixate on a cross corresponding to the location of the treble clef on the next staff. When the staff appeared, the participant started sight-reading the score immediately. Despite being advised to refrain from repeating notes when making mistakes, participants were permitted to play in a natural manner. After playing each excerpt, the participant indicated the level of perceived complexity of the musical score on a Likert scale ranging from 1 (*very easy*) to 5 (*very difficult*) and whether they already knew the excerpt or not by tapping 1 (YES) or 2 (NO) on a button box designed for the experiment. On average, the whole session lasted between 45 and 60 min.

## Results

### Data preparation

To ensure that the task aligned with a typical sight-reading task, we verified that the musicians were only familiar with a limited number of musical scores. On average, the musicians were familiar with 3.19 excerpts out of 68 (*SD* = 3.54), which is <5% of the scores. Given this the low rate of familiar scores, data analysis did not exclude any trials associated with a familiar musical piece.

To ensure that the scores were adapted to the students' levels and were sufficiently challenging for more advanced musicians, we verified the level of the perceived complexity. The musicians perceived globally a medium complexity of the excerpts as shown in Table 1.

**Table 1:** Mean (and standard deviation) perceived complexity according to the level of expertise and the type of score (CL = Classical Music Scores; CO = Contemporary Music Scores; ALL).

	**Perceived complexity**		
**Level**	**CL**	**CO**	**ALL**
N1	2.40 (1.12)	3.07 (1.15)	2.62 (1.17)
N2	1.79 (0.82)	2.59 (1.03)	2.05 (0.97)
N3	2.09 (0.92)	3.75 (1.11)	2.52 (1.14)
N4	1.74 (0.81)	3.02 (1.06)	2.21 (1.10)
N5	1.67 (0.84)	2.77 (1.21)	2.08 (1.12)

**Figure 4:**
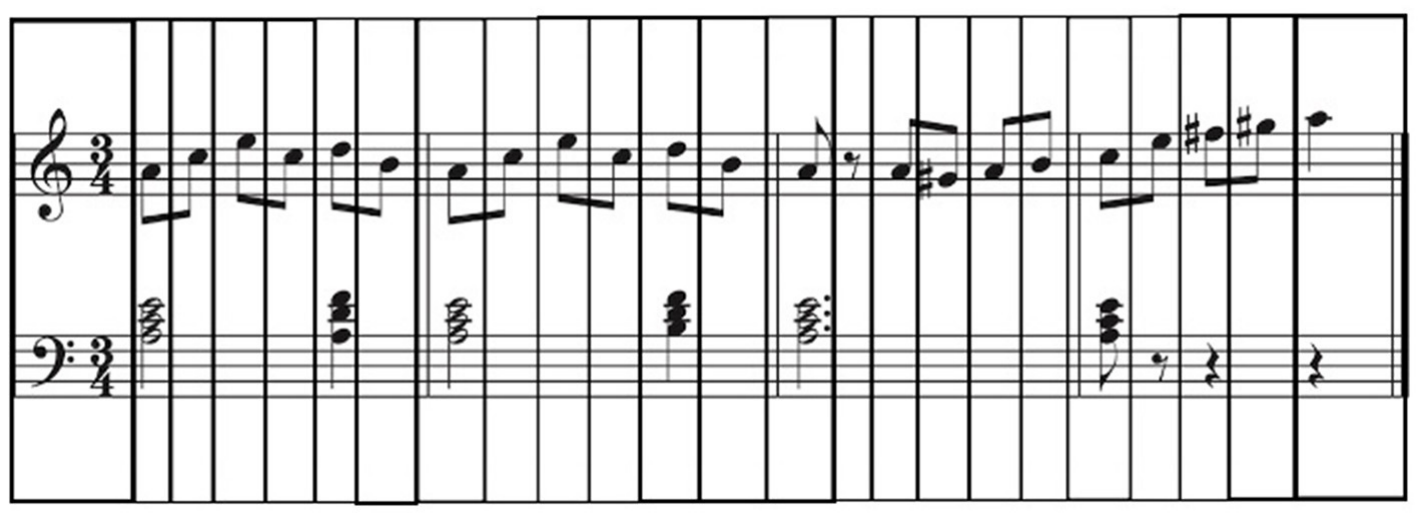
Example of a score with areas of interest.

For collecting the eye-tracking variables from the score (at a threshold of 80 ms considered as an eye fixation), each score was divided into areas of interest (AOIs). An initial AOI included key signatures, time signatures, and further AOIs were related to events (i.e., notes, chords, or rests; Figure 4). The criterion employed for designing different AOIs was that all notes occurring visually simultaneously were included in the same AOI.

### Eye-movement measures

Twenty-four visual measures (Table 2) were extracted (e.g., number of progressive fixations, number of regressive fixations, pupil size, blinks, first-pass fixations, second-pass fixations) from Data Viewer (EyeLink^TM^) for each of the 68 excerpts. The detection of those visual measures, including blinks, was made by the detection methods of Data Viewer software.

**Table 2:** Indicators collected from eye tracking (visual measures), performance (performance measures), and pre- and posttest (subjective measures) to train the SVM.

**Visual measures**	**Performance measures**	**Subjective measures**
AVERAGE_FIXATION_DURATION	EHS	COD
FIRST_PASS_FIX_TOTAL	PLAY_DURATION	CORSI
FIRST_PASS_FIXATION	PLAY_DURATION_NOTE	DIGIT_SPAN_AVERAGE
FIRST_PASS_FIXATION_by_AOI	TEMPO_BPM	PERCEIVED_COMPLEXITY
FIRST_PASS_FIXATION_NOTE	VELOCITY	
FIXATIONS_DURATION_by_note	NUMBER_AOI_CORRECT_TOTAL	
NUMBER_OF_BLINK	%_CORRECT_AOI	
NUMBER_OF_BLINK_NOTE	%_ERRONEOUS_AOI	
NUMBER_OF_FIXATIONS	LATENCY	
NUMBER_OF_FIXATIONS_NOTE	NUMBER_NOTES_WRONG_TOTAL	
NUMBER_OF_PROGRESSIVE_FIXATIONS		
NUMBER_OF_PROGRESSIVE_FIXATIONS_NOTE		
NUMBER_OF_REFIXATIONS		
%_OF_REFIXATIONS		
NUMBER_OF_REFIXATIONS_NOTE		
NUMBER_OF_REGRESSIVE_FIXATIONS		
NUMBER_OF_REGRESSIVE_FIXATIONS_NOTE		
PUPIL_VARIATION		
SECOND_PASS_FIX_TOTAL		
SECOND_PASS_FIXATION		
SECOND_PASS_FIXATION_by_AOI		
SECOND_PASS_FIXATION_NOTE		
SUM_OF_FIXATIONS		
SUM_OF_FIXATIONS_NOTE		

### Performance measures

Ten performance measures (Table 2) were collected from Reaper^T^ software as, for example, play duration (total time to play the score), velocity, latency (before playing the first note), play duration by note, or computed from the data as, for example, EHS, percentage of incorrect AOIs (false notes), and tempo (chosen by the participant). Sight-reading accuracy was evaluated as follows: An AOI was considered correct when all its component elements were correctly played. The proportion of correct and incorrect AOIs played was measured for each score. The sight-reading tempo was evaluated. In the present study, pianists performed without tempo constraint, and the global tempo chosen by the participant was quantified in beats per minute (bpm) for each score. The tempo was quantified with the ratio of the time taken to play the score in milliseconds and the number of beats for each score. Then, by dividing 60,000 by this value, we obtained the chosen tempo in bpm. EHS, the distance between the eye fixating a note on the score and the note played on the piano, was measured using the distance-in-music-unit method (Perra et al., 2021). The EHS has a multimodal dimension and can be used to evaluate the performance aspect of sight-reading (Truitt et al., 1997). It should be noted that, although the EHS encompasses a strong visual aspect, we have chosen to use it as a performance measure here. Nevertheless, this choice does not affect the analysis as all the measures will be computed together by the SVM.

### Subjective measures

Four subjective measures (Table 2) were collected from the cognitive pretests (Digit Span subtest, Coding subtest, and Corsi Block tapping test) as well as the assessment of the perceived complexity following each musical score execution on piano.

All variables were computed independently of their belonging to a specific subgroup. Thus, the diverse attributes of the musicians were taken into account and included in the SVM, along with the eye-tracking variables. Consequently, we trained our SVM on the 38 measures: 24 visual measures, 10 performance measures, and four subjective measures collected while musicians sight-read classical and contemporary music scores. Those measures are detailed in Table 2. Although some measures may be redundant or correlated, we included all of them because the aim of this study is precisely to find out which of the numerous measures collected are the most relevant for distinguishing between the five levels of expertise. The operational definition of each variable is presented Appendix 2.

### SVM implementation

We used the Python machine learning module scikit-learn II to implement the SVM. As the 38 measures had different scales of values, we normalized the data with *StandardScaler()* function, which applied the equation *Z* = (*x* – *u*)/*s*.

In the present study, our objective was to classify 68 pianists into five levels ranging from 1 to 5 (Level 1 = first cycle; Level 2 = second cycle; Level 3 = third cycle; Level 4 = CPES, and Level 5 = CNSM or professional musicians). This hierarchical ranking necessitated using binary classifiers to address ordinal classification problems, where classes are ordered and hierarchical (Frank and Hall, 2001). In this regard, the model was trained to predict shifts between classes rather than specific classes. Therefore, we applied this method by dividing our prediction process into four similar steps: predicting levels 1 and 2 against levels 3, 4, and 5; then refining predictions by comparing level 1 against level 2 and level 3 against levels 4 and 5; and so forth. To evaluate the predictive ability of our model, we divided the participants into two groups for the two phases of our algorithm (learning and testing).

To mitigate overfitting, which occurs when a model overly adapts to the training data, we allocated separate participants for the training and testing phases of the algorithm. Specifically, participants included in the training phase were excluded from the testing phase. In our study, participants were divided such that 70% (48 participants) were used for training and 30% (20 participants) were used for testing.

To maximize the model, we tested its generalization ability to make accurate predictions on new data (rather than on the data it was trained on). As the data were collected during the performance of classical and contemporary music scores, we trained our model on data derived from the performance of classical scores, contemporary scores, and a mix of both types. This provided us with three distinct data sets for training. We also used these combinations for the testing phase. Thus, we obtained nine different combinations to assess the generalization capacity of our model (Table 3). We got three performance indices for each of the four classification phases of our model applied to the nine possible data intersections.

**Table 3:** Results from the data sets used for the training and testing phases with accuracy (ACC) and the area under the ROC curve (AUC) indicators (in bold values corresponding to the probability threshold >0.70).

	**{1–2} vs. {3–5}**		**1 vs. 2**		**3 vs. {4–5}**		**4 vs. 5**	
	**AUC**	**ACC**	**AUC**	**ACC**	**AUC**	**ACC**	**AUC**	**ACC**
All/All	**0.89**	**0.79**	0.49	0.5	**0.75**	**0.71**	0.52	0.48
All/CL	**0.89**	**0.82**	0.49	0.5	**0.75**	0.68	0.48	0.43
All/CO	**0.89**	**0.79**	0.48	0.5	**0.73**	0.68	0.58	0.48
CL/CL	**0.9**	**0.79**	0.47	0.5	**0.79**	**0.74**	0.43	0.43
CL/CO	**0.89**	**0.76**	0.45	0.5	**0.78**	0.68	0.52	0.65
CL/All	**0.9**	**0.76**	0.48	0.5	**0.78**	**0.76**	0.46	0.48
CO/CL	**0.89**	**0.82**	0.52	53	**0.74**	0.68	0.44	0.35
CO/CO	**0.89**	**0.79**	0.54	0.47	**0.73**	**0.71**	0.56	0.52
CO/All	**0.89**	**0.85**	0.52	0.53	**0.74**	0.68	0.51	0.35

These results demonstrate the performance of the model across five levels and different combinations of training and testing data sets, providing insights into its generalization capabilities.

CL/CL, Training: Classical Music Scores, Testing: Classical Music Scores; CO/CO, Training: Contemporary Music Scores, Testing: Contemporary Music Scores; ALL/ALL, Training: Mixed Classical and Contemporary Music Scores, Testing: Mixed Classical and Contemporary Music Scores; CL/CO, Training: Classical Music Scores, Testing: Contemporary Music Scores; CL/ALL, Training: Classical Music Scores, Testing: Mixed Classical and Contemporary Music Scores; CO/CL, Training: Contemporary Music Scores, Testing: Classical Music Scores; CO/ALL, Training: Contemporary Music Scores, Testing: Mixed Classical and Contemporary Music Scores; ALL/CL, Training: Mixed Classical and Contemporary Music Scores, Testing: Classical Music Scores; ALL/CO, Training: Mixed Classical and Contemporary Music Scores, Testing: Contemporary Music Scores.

To evaluate the effectiveness of our model we used the accuracy (ACC) and the area under the Receiver Operating Characteristic (ROC) curve (AUC) indicators. The ACC represents the ratio of correct predictions to the total number of predictions. Therefore, the higher this ratio, the better the precision, theoretically indicating better model performance. The AUC involves the concepts of true positives and false positives. A prediction is considered a true positive when the expected result is 1 and the prediction is also 1 and a true negative when the expected result is 0 and the prediction is also 0. False positives and false negatives occur when the prediction differs from the expected result. By adjusting the decision threshold, we can calculate several ratios based on the rates of true and false predictions, enabling us to plot the ROC curve. The AUC represents the area under this curve and is used as a performance indicator. These characteristics serve to define the predictive capabilities of our model. The closer these indicators are to 1, the more accurate our model's predictions will be, while values closer to 0.5 indicate poorer performance. An AUC value below 0.5 suggests that random guessing would statistically yield better results. Although less common, the AUC is a more revealing indicator of a model's performance, as it reflects not only the accuracy of the predicted labels but also the confidence of the algorithm while making these predictions. This is particularly true when participants are not evenly distributed across classes. Therefore, we use the AUC values as indicators to comment on our results.

**Figure 5:**
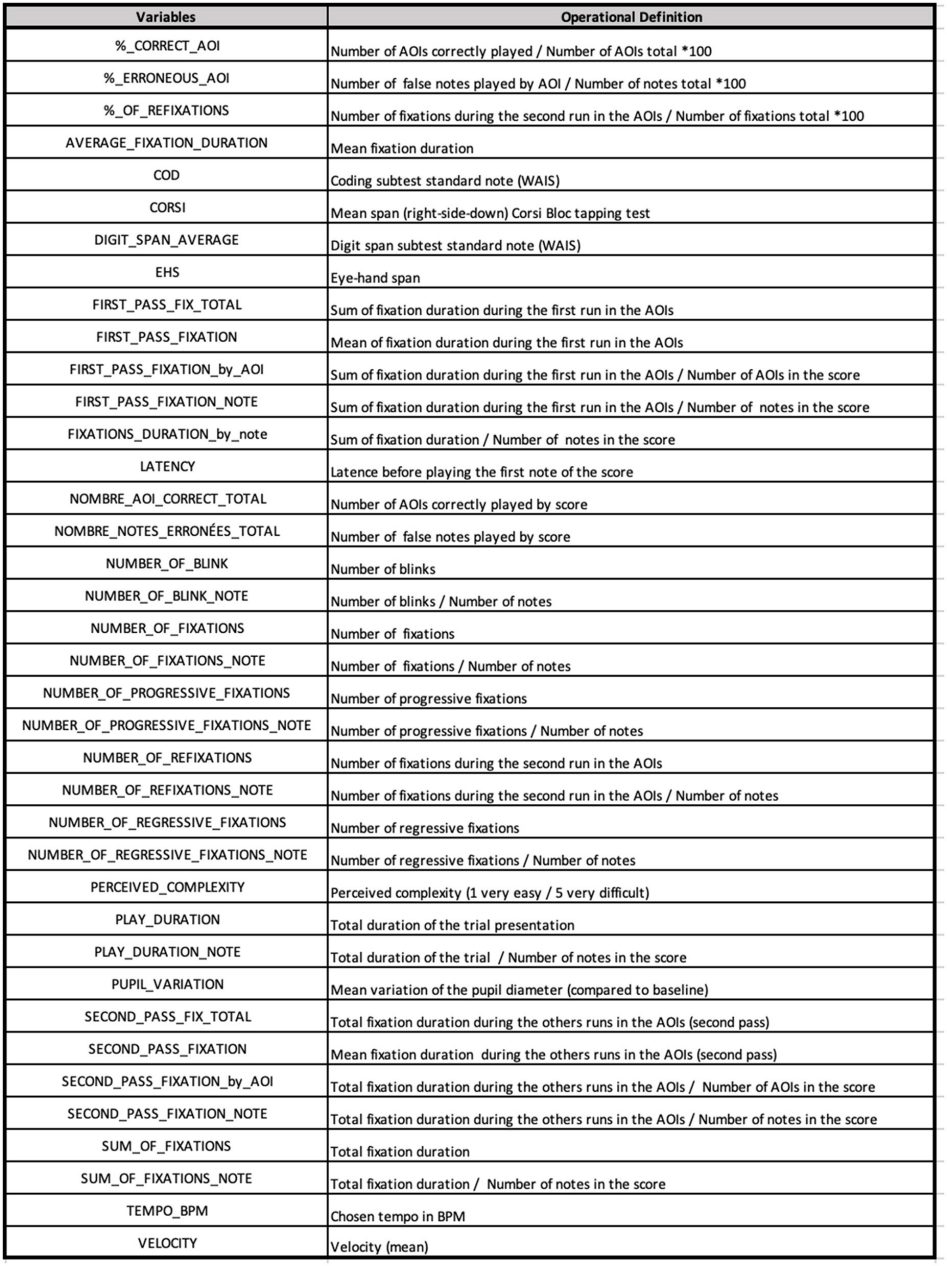
Average area under the curve (AUC) across five levels and different combinations of training and testing data sets.

As we can see (Table 3), regardless of the types of scores [classical [CL], contemporary [CO], all together [ALL]] used for the training and testing phases, we consistently achieve a minimum of 0.89 for classification levels 1 and 2 vs. levels 3, 4, and 5. Similarly, the performance ranges from 0.73 to 0.78 for classification level 3 vs. levels 4 and 5. However, the model's performance is notably low, ranging from 0.45 to 0.54 for classification level 1 vs. 2 and 0.43 to 0.56 for classification level 4 vs. level 5. Figure 5 presents the average AUC related to those classifications across five levels and nine training/testing data sets (classical, contemporary, and all together).

In this first step, SVM used 38 measures (decision variables) to learn and predict the level of 68 pianists across five levels. Our second aim was to find out which of these measures may be crucial in revealing the level of expertise and determining the best predictors for classifying expertise during sight-reading of scores. For this purpose, we created (a) a *UnivariateSVM()* function to execute the classification process using a single decision variable, iteration after iteration, for each of the variables, which allowed us to obtain a performance index for each variable, and (b) a *RecursiveFeatureElimination()* function to remove one decision variable at each iteration. The variable removed was the least useful variable for prediction, determined by comparing the SVM feature coefficients. Therefore, at the end of the execution, we obtained a single variable, the most useful variable for prediction.

Running *UnivariateSVM()* function on a cross section of classical music scores for learning and testing (Table 4) allowed the identification of the performance index for each variable.

**Table 4:** Performance index (AUC value) for each variable after execution of UnivariateSVM() function on classical data (in bold values corresponding to the probability threshold > 0.70).

**Measures**	**{1–2} vs. {3–5}**	**1 vs. 2**	**3 vs. {4–5}**	**4 vs. 5**
%_CORRECT_AOI	0.58 (0.09)	0.52 (0.14)	0.55 (0.14)	0.57 (0.08)
%_ERRONEOUS_AOI	0.66 (0.05)	0.52 (0.14)	0.49 (0.06)	0.60 (0.08)
%_OF_REFIXATIONS	**0.82 (0.06)**	0.69 (0.12)	0.63 (0.19)	0.46 (0.10)
AVERAGE_FIXATION_DURATION	**0.80 (0.08)**	**0.72 (0.08)**	0.55 (0.16)	0.42 (0.12)
COD	0.48 (0.12)	0.37 (0.16)	0.49 (0.18)	0.42 (0.11)
CORSI	0.50 (0.13)	**0.73 (0.15)**	0.66 (0.14)	0.39 (0.16)
DIGIT_SPAN_AVERAGE	0.53 (0.16)	0.59 (0.25)	0.40 (0.10)	0.47 (0.12)
EHS	0.57 (0.08)	0.49 (0.07)	0.53 (0.08)	0.48 (0.07)
FIRST_PASS_FIX_TOTAL	0.69 (0.08)	0.46 (0.12)	0.67 (0.10)	0.37 (0.12)
FIRST_PASS_FIXATION	0.55 (0.14)	0.47 (0.10)	0.46 (0.05)	0.51 (0.10)
FIRST_PASS_FIXATION_by_AOI	0.69 (0.08)	0.41 (0.11)	0.68 (0.10)	0.37 (0.12)
FIRST_PASS_FIXATION_NOTE	0.60 (0.13)	0.50 (0.11)	0.49 (0.09)	0.50 (0.12)
FIXATIONS_DURATION_by_note	**0.86 (0.06)**	**0.74 (0.11)**	**0.75 (0.13)**	0.39 (0.12)
LATENCY	**0.73 (0.06)**	**0.78 (0.10)**	0.60 (0.17)	0.56 (0.15)
NUMBER_AOI_CORRECT_TOTAL	0.56 (0.08)	0.52 (0.13)	0.59 (0.08)	0.59 (0.06)
NUMBER_NOTES_WRONG_TOTAL	**0.77 (0.05)**	0.47 (0.16)	0.63 (0.04)	0.61 (0.06)
NUMBER_OF_BLINK	**0.84 (0.04)**	**0.71 (0.12)**	0.47 (0.09)	0.60 (0.10)
NUMBER_OF_BLINK_NOTE	**0.84 (0.04)**	**0.71 (0.12)**	0.49 (0.17)	**0.71 (0.10)**
NUMBER_OF_FIXATIONS	**0.84 (0.06)**	0.68 (0.14)	**0.76 (0.11)**	0.50 (0.14)
NUMBER_OF_FIXATIONS_NOTE	**0.83 (0.06)**	0.68 (0.14)	**0.76 (0.11)**	0.51 (0.15)
NUMBER_OF_PROGRESSIVE_FIXATIONS	**0.75 (0.07)**	0.60 (0.10)	0.67 (0.10)	0.44 (0.07)
NUMBER_OF_PROGRESSIVE_FIXATIONS_NOTE	**0.77 (0.07)**	0.60 (0.11)	0.69 (0.12)	0.42 (0.09)
NUMBER_OF_REFIXATIONS	**0.84 (0.06)**	**0.70 (0.13)**	**0.74 (0.12)**	0.49 (0.12)
NUMBER_OF_REFIXATIONS_NOTE	**0.84 (0.06)**	0.69 (0.13)	**0.74 (0.12)**	0.49 (0.12)
NUMBER_OF_REGRESSIVE_FIXATIONS	**0.78 (0.06)**	0.67 (0.10)	**0.73 (0.08)**	0.52 (0.07)
NUMBER_OF_REGRESSIVE_FIXATIONS_NOTE	**0.79 (0.06)**	0.68 (0.10)	**0.75 (0.09)**	0.53 (0.07)
PERCEIVED_COMPLEXITY	0.66 (0.06)	0.63 (0.11)	0.55 (0.10)	0.42 (0.09)
PLAY_DURATION	**0.87 (0.06)**	**0.77 (0.12)**	**0.74 (0.11)**	0.60 (0.14)
PLAY_DURATION_NOTE	**0.87 (0.06)**	**0.77 (0.12)**	**0.74 (0.11)**	0.60 (0.14)
PUPIL_VARIATION	0.62 (0.08)	0.45 (0.13)	0.52 (0.11)	0.45 (0.09)
SECOND_PASS_FIX_TOTAL	**0.86 (0.06)**	**0.74 (0.12)**	**0.74 (0.13)**	0.44 (0.11)
SECOND_PASS_FIXATION	**0.70 (0.05)**	0.65 (0.05)	0.45 (0.10)	0.54 (0.08)
SECOND_PASS_FIXATION_by_AOI	**0.85 (0.06)**	**0.74 (0.11)**	**0.74 (0.12)**	0.45 (0.10)
SECOND_PASS_FIXATION_NOTE	**0.72 (0.06)**	0.67 (0.06)	0.59 (0.10)	0.54 (0.10)
SUM_OF_FIXATIONS	**0.86 (0.06)**	**0.74 (0.11)**	**0.75 (0.13)**	0.39 (0.12)
SUM_OF_FIXATIONS_NOTE	**0.86 (0.06)**	**0.74 (0.11)**	**0.75 (0.13)**	0.39 (0.12)
TEMPO_BPM	**0.77 (0.05)**	0.68 (0.10)	0.56 (0.09)	0.52 (0.12)
VELOCITY	0.49 (0.11)	0.35 (0.11)	0.44 (0.08)	0.49 (0.07)

For predicting the level of expertise successfully (with a probability threshold > 0.70) between groups 1, 2 vs. 3, 4, 5, the model identified 24 among 38 variables. Within these variables, 18 were related to visual measures. Notably, among the visual measures identified, eye movements, such as fixation duration by note, the second-pass fixations total, the sum of fixations, the sum of fixations by note, number of blinks, the number of blinks by note, the number of fixations, the number of refixations, and the average fixation duration, exhibited an AUC value exceeding 0.80, indicating a robust performance indicator to classify levels 1 and 2 vs. levels 3, 4, and 5. Additionally, four performance measures were identified: play duration, play duration by note, number of false notes total, tempo (bpm), and latency. Interestingly, none of the subjective measures was identified as a significant predictor in the model.

For predicting the level of expertise successfully between level 1 vs. 2, the univariate model identified 13 among 38 variables (with a probability threshold >0.70). Among these variables, only nine were related to visual measures. Notably, the fixation duration by note, the second-pass fixation total, the second-pass fixation by note, and the sum of fixation exhibited an AUC value exceeding 0.74, indicating a good performance indicator to classify level 1 vs. level 2. Additionally, three performance measures were identified: play duration, play duration by note, and latency and one subjective measure, the Corsi Block tapping test.

For predicting the level of expertise successfully between level 3 vs. levels 4 and 5, the model identified 13 among 38 variables (with a probability threshold >0.70). Within these variables, 11 were related to visual measures. Notably, among those visual measures identified, eye movements such as number of fixations, number of fixations by note, fixation duration by note, number of regressive fixations by note, sum of fixations, sum of fixations by note, exhibited an AUC value exceeding 0.75 indicating a good performance indicator to classify level 3 vs. levels 4 and 5. Additionally, two performance measures were identified: play duration and play duration by note. No subjective measure was identified.

For predicting the level of expertise successfully between level 4 vs. level 5, the model identified only one variable, the number of blinks by note, among 38 variables (with a probability threshold >0.70).

Running *RecursiveFeatureElimination()* function enabled the systematic removal of one decision variable for each iteration, targeting the least informative variable for prediction. The feature selection was performed in a recursive way. We performed prediction on the full matrix, using all features, outputting the coefficient for each SVM (one SVM per split and per binary classification). We took the absolute value of the coefficients averaged across split and then took the maximum across the prediction task to obtain an importance measure for each coefficient. The feature with the smallest importance measure was removed, and the prediction was performed once again without this feature. The features were removed iteratively using this procedure until only one feature remained. The removed features and the corresponding AUC are given (Table A1). The variables at the top were the first ones to be removed and can be considered the least important for the SVM. The variables at the bottom were the last to be removed and are therefore the most informative for the SVM. The last one to be removed was the sum of fixations by note. Even if the performance did not decrease a lot each time a variable was removed, at the end of the completion of the process, the analysis including 38 variables revealed that the four most relevant variables related to the level of expertise were the sum of fixations by note, the number of blinks, the number of fixations and the average fixation duration, the most relevant being sum of fixations by note.

Finally, to assess the model's ability to generalize, we compared the results of the univariate SVM on two data sets. Running *UnivariateSVM()* function, the first data set was obtained by training and testing on classical scores, which serves as a reference since it was tested on the same type of data it was trained on. The second data set was obtained by training on classical scores and testing on contemporary scores. As we can see (Table A2) the results are relatively similar between the two data sets, with each variable obtaining broadly identical performance indices (AUC value) across different level comparisons. Thus, the model presents a strong ability to generalize.

To further refine the identification of the best predictors for distinguishing level 1 from level 2 and level 3 from levels 4 and 5 we opted to reduce the number of measures. We trained our SVM on four preselected eye-tracking (visual) measures, average fixation duration, number of fixations, number of blinks, and number of regressive fixations, across the three data sets and their different combinations of training/testing across five levels. Indeed, a manual selection of relevant variables was conducted before the automatic selection to avoid bias from the latter. Manually selecting variables beforehand provides a more reliable performance assessment, untainted by the outcomes of automatic selection. We manually identified a subset of visual variables that were relevant, complementary, and with no redundancy. Interestingly, among these *a priori* chosen variables based on previous research (e.g., Drai-Zerbib and Baccino, 2018), the average fixation duration, the number of fixations, and the number of blinks were also identified by the SVM as the most informative variables (after the most important one which is the sum of fixations by note). As illustrated in Table 5, the corresponding AUC indicates that using only these four variables produced satisfactory outcomes for comparisons between level 1 and level 2 and between level 3 and levels 4 and 5. The performance was found to be fairly robust when adding or removing a variable. However, the model, once again, failed to predict the level of expertise between groups 4 and 5, with a decreased probability threshold (<0.45). However, regardless of the types of scores (CL, CO, and ALL) used for the training and testing phases, the model better predicted the level of expertise between levels 1 and 2, with an increased probability threshold (>0.74) similar to the one obtained to distinguish level 3 from levels 4 and 5 with the 38 measures. Furthermore, the model produced the same results for the training and testing phases with 4 visual measures compared to 38 measures for the other comparisons. Thus, this result confirms that the proposed model has a high generalization capability and can be effectively applied to different comparison levels in the three data sets (CL, CO, and ALL), providing similar results.

**Table 5:** Results (AUC value) from the data sets used for the training and testing phases with four visual measures (in bold values corresponding to the probability threshold >0.70).

	**Four measures**	
All/All	1–2 vs. 3–5	**0.87 (0.05)**
	1 vs. 2	**0.76 (0.11)**
	3 vs. 4–5	**0.71 (0.13)**
	4 vs. 5	0.45 (0.13)
All/CL	1–2 vs. 3–5	**0.88 (0.05)**
	1 vs. 2	**0.76 (0.11)**
	3 vs. 4–5	**0.73 (0.14)**
	4 vs. 5	0.45 (0.14)
All/CO	1–2 vs. 3–5	**0.86 (0.05)**
	1 vs. 2	**0.74 (0.11)**
	3 vs. 4–5	0.68 (0.13)
	4 vs. 5	0.44 (0.12)
CL/All	1,2 vs. 3–5	**0.87 (0.05)**
	1 vs. 2	**0.75 (0.12)**
	3 vs. 4–5	**0.71 (0.13)**
	4 vs. 5	0.44 (0.11)
CL/CL	1–2 vs. 3–5	**0.88 (0.05)**
	1 vs. 2	**0.76 (0.12)**
	3 vs. 4–5	**0.73 (0.14)**
	4 vs. 5	0.44 (0.11)
CL/CO	1–2 vs. 3–5	**0.86 (0.05)**
	1 vs. 2	**0.74 (0.12)**
	3 vs. 4–5	0.68 (0.13)
	4 vs. 5	0.44 (0.11)
CO/All	1–2 vs. 3–5	**0.87 (0.05)**
	1 vs. 2	**0.77 (0.09)**
	3 vs. 4–5	**0.70 (0.14)**
	4 vs. 5	0.45 (0.14)
CO/CL	1–2 vs. 3–5	**0.88 (0.05)**
	1 vs. 2	**0.77 (0.10)**
	3 vs. 4–5	**0.72 (0.14)**
	4 vs. 5	0.45 (0.15)
CO/CO	1–2 vs. 3–5	**0.86 (0.06)**
	1 vs. 2	**0.75 (0.09)**
	3 vs. 4–5	0.67 (0.13)
	4 vs. 5	0.45 (0.13)

These results demonstrate the performance of the model trained with four eye-tracking measures (average fixation duration, number of fixations, number of blinks, and number of regressive fixations) across five levels and different combinations of training and testing data sets.

CL/CL, Training: Classical Music Scores, Testing: Classical Music Scores; CO/CO, Training: Contemporary Music Scores, Testing: Contemporary Music Scores; ALL/ALL, Training: Mixed Classical and Contemporary Music Scores, Testing: Mixed Classical and Contemporary Music Scores; CL/CO, Training: Classical Music Scores, Testing: Contemporary Music Scores; CL/ALL, Training: Classical Music Scores, Testing: Mixed Classical and Contemporary Music Scores; CO/CL, Training: Contemporary Music Scores, Testing: Classical Music Scores; CO/ALL, Training: Contemporary Music Scores, Testing: Mixed Classical and Contemporary Music Scores; ALL/CL, Training: Mixed Classical and Contemporary Music Scores, Testing: Classical Music Scores; ALL/CO, Training: Mixed Classical and Contemporary Music Scores, Testing: Contemporary Music Scores.

## Discussion

This study used an advanced machine learning technique to classify musicians according to their level of expertise by analyzing their eye movements synchronized with their playing behavior during sight-reading of classical and contemporary scores. We used an SVM to, first, investigate whether the level of expertise of the musicians could be reliably inferred from eye movements, performance, and subjective measures during music reading and, second, exhibit the most relevant predictors for classifying levels/groups of expertise in music reading. Our SVM was trained and tested on eye movement, performance, and subjective measures from 68 pianists at five levels of music expertise (first cycle to professional musicians), sight-reading classical and contemporary music excerpts. The SVM analysis included 24 visual measures (e.g., number of progressive fixations, number of regressive fixations, pupil size, first-pass fixations, and second-pass fixations), ten performance measures (e.g., eye-hand span, percentage of incorrect notes per AOI, tempo, play duration, and velocity), and four subjective measures (i.e., perceived complexity and cognitive skills). Those different types of variables were all included in the SVM and computed together independently of their belonging to a subgroup. Musical sight-reading engages mechanisms and concepts related to expertise, encompassing the management of multimodal information, the acquisition of reading skills, auditory processing, motor skills, memory, and attention. Investigating the construction of expertise in musical sight-reading can provide insights into the general mechanisms underlying expertise development.

The results indicated the robust classification accuracy achieved by the model. It effectively classified different levels of expertise based on eye movements, performance, and subjective measures. The model exhibited a very high accuracy of prediction in classifying lower level musicians (1 and 2) from other levels (3, 4, and 5), as well as a highly accurate prediction in classifying medium-level musicians (3) compared to higher level musicians (4 and 5). However, it demonstrated comparatively poorer performance when comparing levels 4 and 5 and levels 1 and 2 when tacking all the 38 variables to run the SVM. This suggests that distinguishing between lower levels (cycles 1 and 2) based on eye movements, performance, and subjective measures may be challenging due to ongoing development in music processing competencies and relatively similar eye movements and performance. The Corsi Block tapping test, evaluating visuospatial working memory, proved to be a good classification variable between groups 1 and 2, in particular for classical music data. Thus, the visuospatial span may be a predictor to distinguish the lower levels of expertise in music. Conversely, discerning between higher levels (CPES, CNSM, and professional musicians) was difficult, likely due to a similar elaboration level of expert memory structures developed over years of learning and practice. Their eye movements, performance, and subjective measures were very comparable. Therefore, these results answer positively to our first hypothesis: the level of expertise of the musicians can be reliably inferred from eye movements, performance, and subjective measures during music reading, using SVM. In particular, the model effectively classified three levels of expertise in music sight-reading, non-experts (levels 1 and 2), medium-level musicians (level 3), and experts (CPES, CNSM, and professional musicians).

That a smaller number of subjects may lead to poorer training of the SVMs and therefore to poorer performance is worth noting. However, to validate that the differences in performance were mainly due to the difficulty of separating similar levels 4 and 5 and not to the number of subjects, we trained and tested levels 1 and 2 vs. levels 3, 4, and 5 classifications on only some of the subjects (from 20 to 100%). We observed a drop in performance of only two points (AUC of 0.87 instead of 0.89) when only 20 of the 68 subjects were included. This shows that the performance achieved on the more advanced classifications (0.52 for 4 vs. 5) is related to the similarity of the groups and not to the number of subjects used to train the SVMs. Moreover, the performance of the model trained with only four eye movement variables (i.e., the number of blinks, the average fixation duration, number of fixations, and number of regressive fixations) showed a good classification accuracy to discriminate levels 1 from 2 and level 3 from levels 4 and 5, with a performance that is quite robust to the addition or removal of a variable. However, this is not the case for level 4 vs. level 5.

The results also provided the relevant predictors for classifying musical expertise. In the first step, the model identified relevant variables for classifying different levels. A high number of variables were identified (24) to distinguish lower (levels 1 and 2) from other levels of expertise (level 3, CPES, CNSM, and professional musicians). These predictors predominantly comprised visual measures (e.g., the fixation duration by note, the second-pass fixations total, the sum of fixations, the sum of fixations by note, the number of blinks, the number of blinks by note, the number of fixations number of refixations, and the average fixation duration) and performance measures [e.g., play duration, play duration by note, the number of false notes total, tempo [bpm], and latency], as none of the subjective measures was identified in this classification. Moreover, for prediction between level 3 (medium level) and higher levels (CPES, CNSM, and professional musicians), the model identified a lower number of variables (13). These mainly comprised visual measures (e.g., number of fixations, number of fixations by note, fixation duration by note, number of regressive fixations by note, sum of fixations, and sum of fixations by note) and performance measures (e.g., play duration and play duration by note). Again, no subjective measure was identified for this classification. Furthermore, the model identified 13 variables for prediction between levels 1 and 2. These predictors were mainly visual measures (e.g., fixation duration by note, second-pass fixation total, second-pass fixation by note, and sum of fixations) and performance measures (e.g., play duration, play duration by note, and latency). Interestingly, a subjective measure, the Corsi Block tapping test, evaluating visuospatial working memory, was identified in this classification. This result confirms that the visuospatial memory span can serve as a valuable indicator for classifying musical expertise between levels 1 and 2. This is particularly evident in the current study when musicians are instructed to play naturally while refraining from repeating notes upon making mistakes. Interestingly, among the 38 variables considered, the model only identified one predictor, the number of blinks by note, to distinguish CPES (level 4) from CNSM and professional musicians (level 5). As blink rate is an effective measure of mental workload (Da Tao et al., 2019) and blink rate decreases as mental workload increases across various tasks and domains (Holland and Tarlow, 1972), this suggests that the mental workload during music sight-reading may be the only difference between those two higher levels of musicians. Thus, blink may be a very useful measure to distinguish those levels of expertise.

In the second step, the SVM model provided the most relevant predictors among the 38 measures. The four most relevant measures identified were visual variables such as the sum of fixations by note, the number of blinks, the number of fixations, and the average fixation duration. Therefore, these results affirmatively answer our second inquiry: the SVM can provide relevant predictors within 38 measures to distinguish levels and, in particular, identified the four most relevant variables for classifying different levels of expertise. Furthermore, these results align with previous research showing the importance of average fixation duration and number of fixations to distinguish levels of expertise. Many studies have consistently shown that an expert memory, built over years of practice, enables expert musicians to read and perform faster musical scores compared to non-experts. Experts exhibit fewer fixations (Drai-Zerbib and Baccino, 2014; Perra et al., 2024; Waters et al., 1997) and shorter fixation durations (Drai-Zerbib and Baccino, 2005, 2014, 2018; Drai-Zerbib et al., 2012; Goolsby, 1994; Penttinen et al., 2013; Waters et al., 1997; Waters and Underwood, 1998; Perra et al., 2022, 2024) compared to non-experts. Moreover, these results are consistent with previous studies, comparing expert and non-expert musicians, showing that eye movements in sight-reading evolve with the development of musical expertise and may reflect the degree of elaboration of the expert memory structures developed over years of learning and practice (Drai-Zerbib and Baccino, 2018; Penttinen and Huovinen, 2011; Perra et al., 2024). Thus, eye movements in music reading are systematically influenced by the level of expertise. Consequently, the level of expertise of the musicians may be inferred from eye-movement behavior using an SVM. Indicating that the number of blinks could also be a predictive variable of expertise, this study also brings a novel insight regarding expertise. Blinks are quite informative regarding cognitive load, as the number of blinks reduces with the increasing workload dedicated to a task (Brookings et al., 1996). Thus, this suggests that the number of blinks reveals the relationship between the cognitive resources (attention and memory) demanded by a sight-reading task and a musician's ability to allocate those resources can reliably distinguish expertise levels. This result might be linked to an increase in structural processing abilities through the acquisition of music sight-reading expertise, with tonal-specific cues playing a significant role in facilitating efficient eye-movement behavior and making fluent sight-reading easier.

Moreover, SVM proved to be highly reliable, as evidenced by the model's accurate generalization across different types of musical scores. This result is important because we were keen to train our SVM on the 38 measures, including correlated predictors such as for example number of blinks and number of blinks per note, percentage correct AOIs played, and percentage erroneous AOIs played, and one potential issue can be that such predictors can be overweighted in the model (thus, the performance can be affected if many redundant variables are included). Usually, it is prudent to evaluate the impact of high correlations among predictors, especially if performance issues arise. In such cases, dimensionality reduction techniques, like principal component analysis, can be employed to mitigate the effects of multicollinearity before applying an SVM. However, we opted not to take this step as we did not encounter any performance issues. Indeed, our results were highly satisfactory. Currently, we have demonstrated that our SVMs can consistently achieve a minimum accuracy of 0.89 for classification between lower (1–2) and higher (3–5) levels. Similarly, the performance ranges from 0.73 to 0.78 for classification level 3 vs. level 4 and 5. Moreover, in cases of multicollinearity, the model tends to have poor generalization ability and may overfit the data, leading to poor performance on unseen data (Chan et al., 2022). This is not the case in our study. To maximize the model, we tested its generalization ability to ensure accurate predictions on new data rather than solely on the training data. We have shown that our SVMs achieve a similar performance on classical partitions on which they were trained and on contemporary partitions they have never seen. Indeed, the model's performance, when trained and tested on classical scores, was comparable to that achieved when training on classical scores and testing on contemporary scores. This result is also particularly interesting as it suggests that musical expertise may exhibit specificity not only in terms of knowledge, in which the sophistication of knowledge structures would relate to the rules governing a particular activity (Perra et al., 2024), but also in a broader domain-specific context. In other words, it implies that expertise in music reading may extend beyond the type of composition usually performed, encompassing a deeper understanding of the underlying principles and conventions that govern musical performances across various genres. Indeed, previous studies showed that musicians develop an expert memory and activate high-level knowledge structures to generate expectations about the musical structure of the score. In particular, knowledge structures specific to tonal music such as retrieval structures may facilitate the encoding and retrieval of information during music reading (Drai-Zerbib et al., 2012; Ericsson and Kintsch, 1995, 2000). The present study shows that the model was able to generalize with classical scores, composed in a tonal architecture, corresponding to the type of music frequently studied and performed during music education. The model was also able to generalize with contemporary scores, which, even written with the same code (chords and succession of notes), does not respect the rules of tonal music, is less studied during music education. Thus, the model can efficiently learn from a type of score and predict with another type of score. Interestingly, performance measures were not considered the best classifiers (compared to the top ones), even though it might be expected that performance is synonymous with expertise. However, one might consider that the less expert participants were more focused on playing accurately, even if it meant reducing the chosen tempo for these short excerpts. This trade-off could probably explain such a result.

In addition to elucidating the reliability and rationale behind employing SVM for classifying varying levels of expertise in music sight-reading, these findings further substantiate the assertion that expert musicians leverage their prior knowledge and expert memory to sight-read proficiently. These outcomes bear significant theoretical and practical implications for music cognition and pedagogy. Theoretical implications arise from these findings as they offer insights into the development of an expert memory and the reconfiguration of cognitive processes over the course of learning from the first cycle to the professional level. This restructuring becomes evident at the level CPES (as levels 4 and 5 were difficult to distinguish), showing a threshold in the learning of music reading following the entry in CPES, beyond which eye movements and performance no longer diverge, indicating the establishment of expert memory (Ericsson and Kintsch, 1995). The present study once again validated that the number and duration of fixations are both robust indicators of expertise in music reading. Additionally, the number of blinks, reflecting the level of mental workload, may serve as a distinguishing measure, in particular for higher levels of expertise, highlighting the intricate interplay between eye movements and cognitive processing. This study contributes to our understanding of the intricate mechanisms involved in expert music reading, offering valuable insights into the cognitive processes of the musician's brain for researchers, conservatory professors, and musicians. Practical implications arise from these findings as SVM can help to provide an innovative way to assess the level of expertise. By leveraging an SVM for eye-movement analysis, a platform equipped with eye-tracking technology can be developed to assess and refine music-reading expertise across diverse levels. Integrating eye tracking and SVM analysis, this platform would provide an objective assessment of music-reading expertise during sight-reading exercises. Such a tool could assist educators and conservatories in providing the best teaching for students. Based on individual profiles delineated by specific eye movements (e.g., the sum of fixations by note, the number of blinks, the number of fixations, and the average fixation duration) students could be assigned to groups matching their profiles, fostering a harmonious progression in their learning and expertise development. This pedagogical tool would empower educators to make informed decisions regarding the assignment of music reading levels, particularly during pivotal end-of-year or end-of-cycle evaluations. Furthermore, by aligning students' profiles with their respective music-reading levels, tailored exercises can be administered to enrich their learning experience and effectively develop expertise in music. Processing and integrating musical notation according to the level of expertise is a crucial question for music teaching in terms of both training and profiling young musicians to provide them with the most fitted teaching. The present study showed that innovative methods such as machine learning and eye tracking can enhance the understanding of expertise in music reading, and beyond, as music reading offers a unique insight into the research area of expert memory. Music reading involves sequential information processing, wherein the attentional focus continuously shifts to the upcoming note in the reading direction (Rayner, 1998) and involves multisensory information processing (auditory, visual, and motor; Drai-Zerbib and Baccino, 2005, 2014, 2018; Stewart et al., 2003).

## Data Availability

The raw data supporting the conclusions of this article will be made available by the authors, without undue reservation.
