# Supplementary material for: Classifying musical reading expertise by eye-movement analysis using machine learning
Source: Front Cognit. 2024 Aug 30;3:1417011. doi: 10.3389/fcogn.2024.1417011 (PMC13281050; doi:10.3389/fcogn.2024.1417011)
Supplement: Supplementary file 1 [file Data_Sheet_1.docx]

**Appendix 1** Selected excerpts from ecological scores

| **TONAL SCORES** | **ATONAL SCORES** |
| --- | --- |
| Menuette & Aria, Joseph Haydn | Notes et Menottes Vol.1, Claude Baliff |
| Menuette Trio, Joseph Haydn | Notes et Menottes Vol.1, Claude Baliff |
| Polonaise, Jan Ladislav Dussek | Douze Notations, Pierre Boulez |
| Menuette & Aria II, Joseph Haydn | Notes et Menottes Vol.1, Claude Baliff |
| Allegro, Joseph Haydn | Blackbird, Henri Dutilleux |
| Vagabond's song II, Béla Bartok | Hana-Bi, Joe Hisaishi |
| A little song, Dmitri Kabalevsky | In a Landscape, John Cage |
| Air, Wolfgang Amadeus Mozart | In a Landscape, John Cage |
| Andante, Wolfgang Amadeus Mozart | Douze Notations, Pierre Boulez |
| Burleska, Wolfgang Amadeus Mozart | Douze Notations, Pierre Boulez |
| Rondo, Wolfgang Amadeus Mozart | Blackbird, Henri Dutilleux |
| Menuetto, Wolfgang Amadeus Mozart | In a Landscape, John Cage |
| Rigaudon, Johann Ludwig Krebs | Metamorphosis I, John Cage |
| Menuett, Johann Sebastien Bach | Metamorphosis I, John Cage |
| Sarabande, Georg Friedrich Haendel | Strophe I, Claude Baliff |
| Prélude, Georg Friedrich Haendel | Strophe II, Claude Baliff |
| The fair, Karl Czerny | 90+, Elliott Cook Carter |
| Une larme, Modeste Petrovitch Moussorgski | Strophe II, Claude Baliff |
| Sonate N°11, Domenico Cimarosa | Sonate10, Henryk Górecki |
| Six scottish, Ludwig van Beethoven | Kindertanz Nikos Skalkottas |
| Vagabond's song, Béla Bartok | KleinerKanon, Nikos Skalkottas |
| Minuet, Georg Friedrich Haendel | Tango, Nikos Skalkottas |
| Le moine bourru, Robert Schumann | Kleiner Bauernmarsch, Nikos Skalkottas |
| 29° étude, Book 1, Henry Lemoine | Wasserklavier (Ext3), Luciano Berio |
| Prélude, Frédéric Chopin | Wasserklavier (1965), Luciano Berio |
| Op., 37, 10è étude, Book 1, Henry Lemoine |  |
| Sarabande, Arcangelo Corelli |  |
| Fantasia, Georg Philip Telemann |  |
| Marcia, Wolfgang Amadeus Mozart |  |
| Waltzer, Johannes Brahms |  |
| Danse, Carl Czerny |  |
| Le moulin, Théodore Lack |  |
| Courante, Georg Friedrich Haendel |  |
| Menuet II, Johann Sebastien Bach |  |
| Menuet, Wolfgang Amadeus Mozart |  |
| Soldiers’s march, Robert Schumann |  |
| Personal composition 1, Jean-Louis Luzignant |  |
| Personal composition 2, Jean-Louis Luzignant |  |
| Personal composition 3, Jean-Louis Luzignant |  |
| Personal composition 4, Jean-Louis Luzignant |  |
| Personal composition 5, Jean-Louis Luzignant |  |
| Personal composition 6, Jean-Louis Luzignant |  |
| Personal composition 7, Jean-Louis Luzignant |  |

**Appendix 2 Operational definition variables**

**
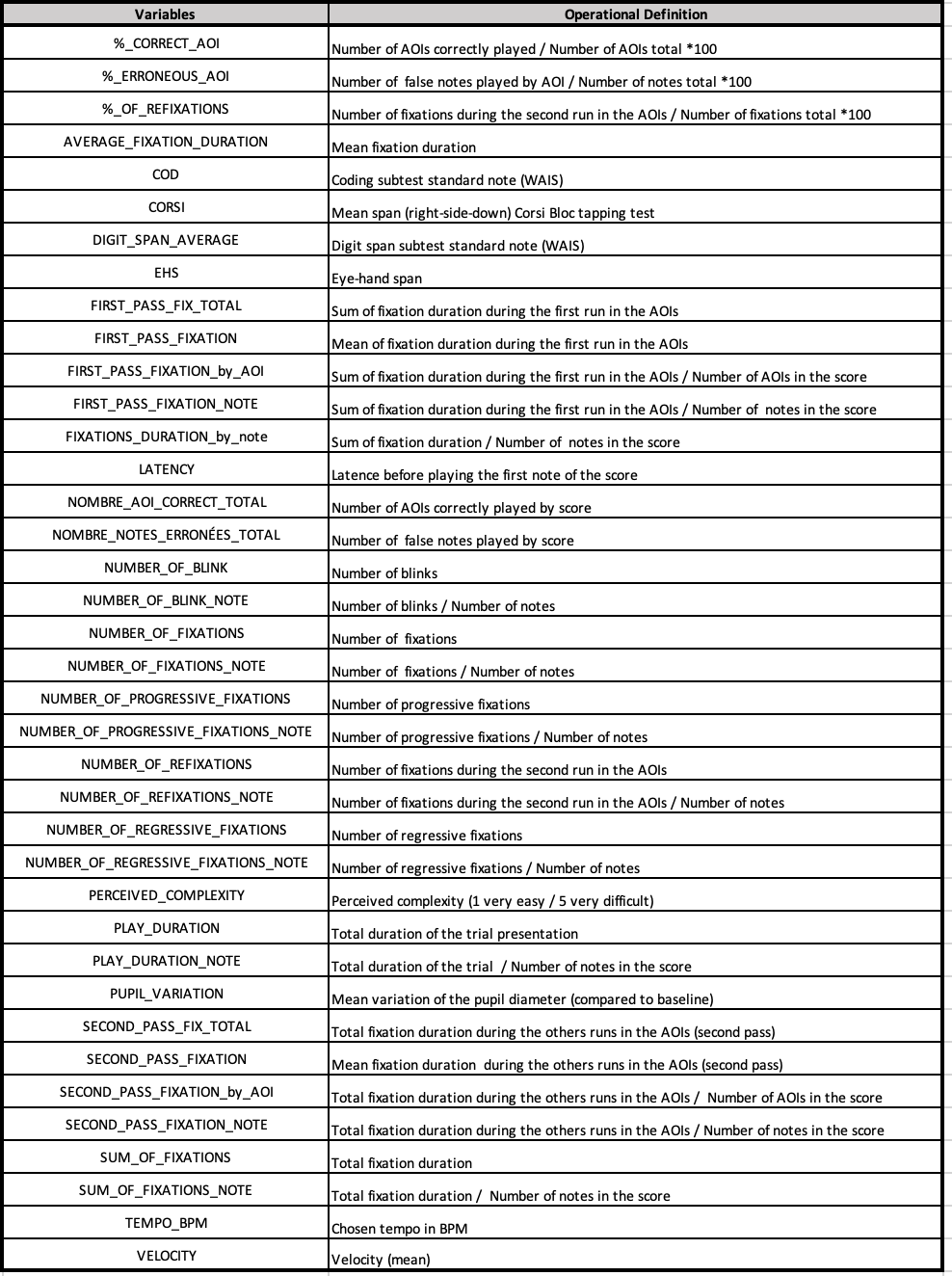
**
