# Supplementary material for: Classifying musical reading expertise by eye-movement analysis using machine learning
Source: Front Cognit. 2024 Aug 30;3:1417011. doi: 10.3389/fcogn.2024.1417011 (PMC13281050; doi:10.3389/fcogn.2024.1417011)

# Annexes

*Table A1 Performance index (AUC value) for each variable (in bold values corresponding to the probability threshold > 0.70). after execution on Classical data of RecursiveFeatureElimination()*

**
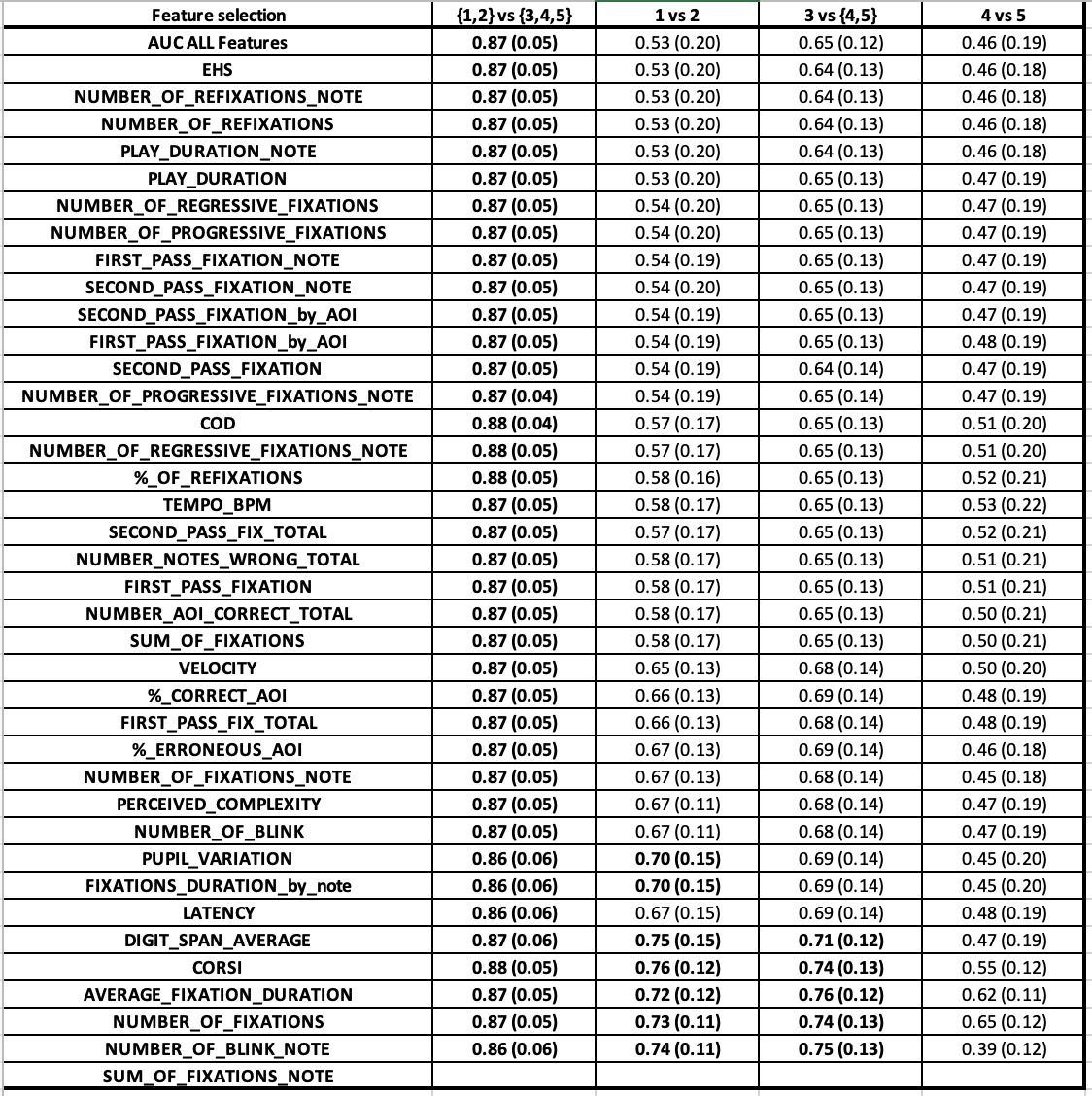
**

*Table A2 Comparison of the results of two datasets (CL/CL, CL/CO). Performance index (AUC value) for each variable after execution of UnivariateSVM() function for training/testing on Classical scores (CL/CL) and training on Classical scores / testing on contemporary scores (CL/CO), (in bold values corresponding to the probability threshold > 0.70).*


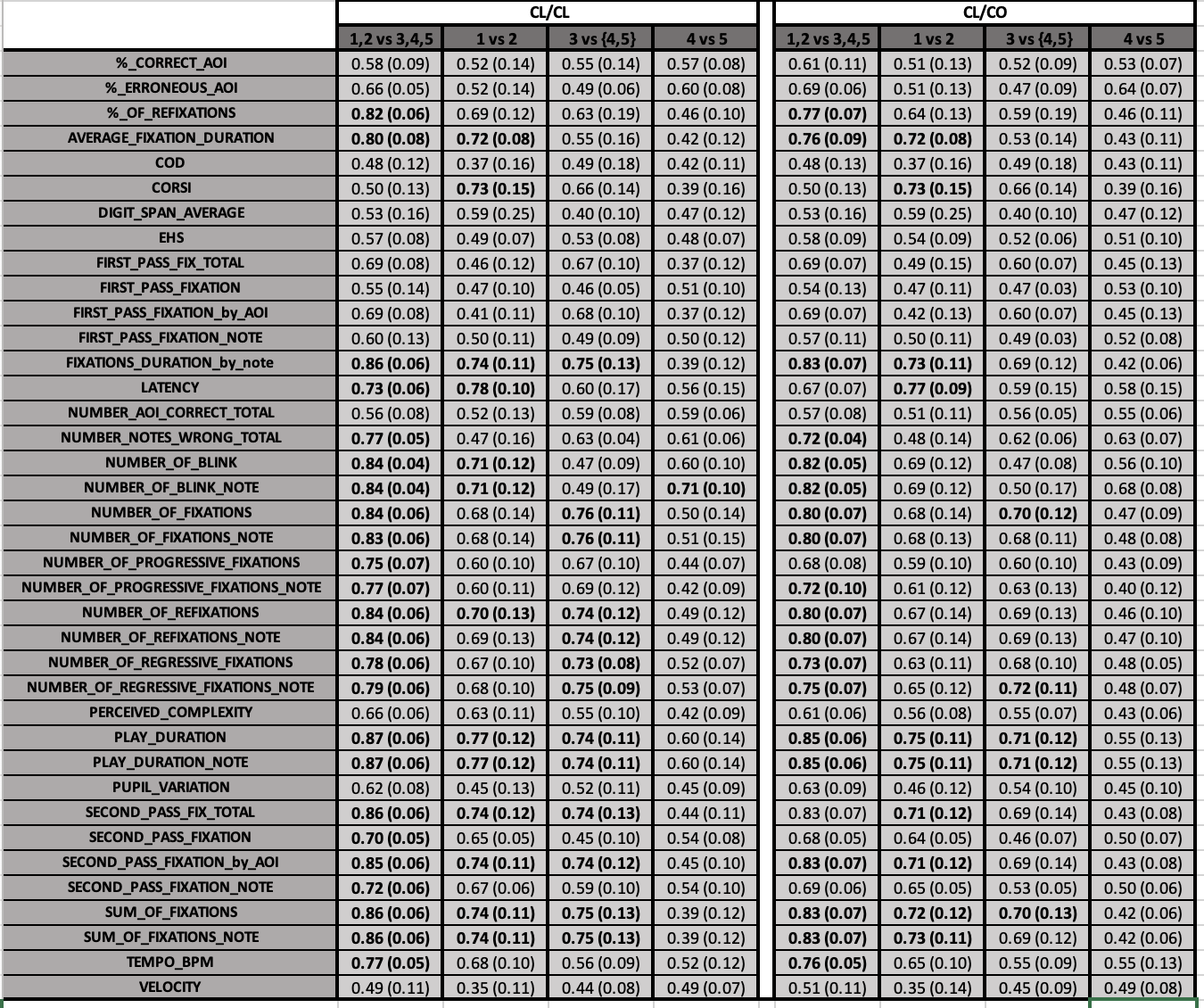

Supplement: Supplementary file 2 [file Table_1.docx]
